# QTL Mapping of Adult Plant Resistance to Stripe Rust in a Doubled Haploid Wheat Population

**DOI:** 10.3389/fgene.2022.900558

**Published:** 2022-05-11

**Authors:** Muhammad Massub Tehseen, Fatma Aykut Tonk, Muzaffer Tosun, Harpinder Singh Randhawa, Ezgi Kurtulus, Izzet Ozseven, Behyan Akin, Ozge Nur Zulfuagaoglu, Kumarse Nazari

**Affiliations:** ^1^ Department of Field Crops, Ege University, Izmir, Turkey; ^2^ Agriculture and Agri-Food Canada, Lethbridge, AB, Canada; ^3^ Turkey-ICARDA Regional Cereal Rust Research Center (RCRRC), Izmir, Turkey; ^4^ Agean Agricultural Research Institute, Regional Cereal Rust Research Center (RCRRC), Izmir, Turkey; ^5^ International Maize and Wheat Improvement Center (IWWIP-Turkey), Ankara, Turkey

**Keywords:** QTL mapping, yellow rust, adult plant resistance, doubled haploid (DH), wheat

## Abstract

Stripe rust caused by *Puccinia striiformis Westend.* f. sp. *tritici*. is a major bread wheat disease worldwide with yield losses of up to 100% under severe disease pressure. The deployment of resistant cultivars with adult plant resistance to the disease provides a long-term solution to stripe rust of wheat. An advanced line from the International Winter Wheat Improvement Program (IWWIP) 130675 (Avd/Vee#1//1-27-6275/Cf 1770/3/MV171-C-17466) showed a high level of adult plant resistance to stripe rust in the field. To identify the adult plant resistance genes in this elite line, a mapping population of 190 doubled haploid (DH) lines was developed from a cross between line 130675 and the universal stripe rust-susceptible variety Avocet S. The DH population was evaluated at precision wheat stripe rust phenotyping platform, in Izmir during 2019, 2020, and 2021 cropping seasons under artificial inoculations. Composite interval mapping (CIM) identified two stable *QTLs QYr.rcrrc-3B.1*, and *QYr.rcrrc-3B.2*, which were detected in multiple years. In addition to these two *QTLs*, five more *QTLs*, *QYr.rcrrc-1B*, *QYr.rcrrc-2A*, *QYr.rcrrc-3A*, *QYr.rcrrc-5A*, and *QYr.rcrrc-7D*, were identified, which were specific to the cropping year (environment). All *QTLs* were derived from the resistant parent, except *QYr.rcrrc-3A*. The significant QTLs explained 3.4–20.6% of the phenotypic variance. SNP markers flanking the *QTL* regions can be amenable to marker-assisted selection. The best DH lines with high yield, end-use quality, and stripe rust resistance can be used for further selection for improved germplasm. SNP markers flanking the QTL regions can aid in identifying such lines.

## Introduction

Wheat stripe (yellow) rust, caused by *Puccinia striiformis* Westend. f. sp. *tritici* (*Pst*), is one of the most important and devastating diseases of common wheat (*Triticum aestivum* L.) around the world (Hovmøller et al., 2011). It remains a significant threat to wheat yield loss, and under severe disease pressure, yield losses of up to 100% are observed (Ali et al., 2014). Stripe rust was historically considered a disease of wheat-growing areas with cool temperatures; however, with the emergence of adapted races to high temperatures and more aggressive races, the disease is now spreading to areas where it was previously considered unfavorable (de Vallavieille-Pope et al., 2012; Muleta et al., 2017; Godoy et al., 2018). Today, the new pathotypes of stripe rust are prevalent from Europe to Australia, Asia, and America and as a result threatens the wheat production on a global scale (Ali et al., 2014).

The continuous occurrence of new stripe rust races requires the identification of new sources of resistance and the deployment of resistant varieties in a timely manner. The conventional approaches for controlling stripe rust include cultural practices like early sowing and crop rotation to avoid infection during the disease infestation period (Boyd 2005). Additionally, fungicide application is also an effective way of controlling stripe rust; however, it is not the most economical and recommended method (Brar et al., 2018). The most effective strategy to control stripe rust outbreaks is the exploitation of genetic resistance and pyramiding of multiple minor and major stripe rust resistance genes conferring seedling and adult plant resistance (APR) (Chen et al., 2014; Tadesse et al., 2014; Muleta et al., 2017; Cobo et al., 2018). Most breeding programs in the world rely on two types of genetic resistance based on major and minor genes (Chen et al., 2014). Genetic resistance due to major genes is termed as a seedling and/or all-stage resistance and is often race-specific and based on the gene for gene hypothesis and is effective throughout a plant’s life (Burdon et al., 2014; Tehseen et al., 2021). However, such resistance in commercial wheat cultivars is often short-lived and is overcome by new races of stripe rust pathogens virulent on the major resistance gene (Boyd 2005; Ellis et al., 2014; Hulbert and Pumphrey 2014), whereas the minor gene resistance is often not expressed until in the later stages of plant life and is commonly referred to as horizontal or adult plant stage resistance (Steele et al., 2001; Boyd 2005). Therefore, many wheat breeding programs consider pyramiding of both seedling and APR genes for enhancing the durability of resistance to multiple prevalent races of stripe rust, hence minimizing yield losses. Due to new emerging races of the stripe rust pathogen virulent to numerous seedling or race-specific genes, the best strategy would be to stack multiple non-race-specific or APR genes in combinations for durable stripe rust resistance (Rajaram 2015). Therefore, although the characterization of seedling resistance genes from highly resistant lines is crucial, the elite breeding lines with multiple adult plant QTLs having high to moderate resistance levels should be considered more important. Elite breeding lines having higher agronomical, biotic, and abiotic stress resistance and end-use quality traits tend to be the ideal candidates for gene mapping as they can be readily used in the ongoing breeding programs.

The bread wheat has a very large genome size; additionally, the allopolyploidy further hampers the progress of mapping new quantitative trait loci (QTLs) and as a result slows down the breeding process (Liu et al., 2021). The whole genome of the common wheat cultivar Chinese Spring was completed 14 years later than some of the other gramineous crops such as rice; thus, it made genetic association comparisons at the whole genome level more complex than other crops (Yu et al., 2002; Wang et al., 2018). Recently, with advances in wheat genome sequencing, multiple versions of the annotated wheat genome have been published consequently accelerating forward genetic research (Clavijo et al., 2017; Zimin et al., 2017; Appels et al., 2018). Today, due to high-throughput sequencing platforms, the development of a large number of high-quality markers is possible, thus facilitating more efficient mapping techniques to analyze a large number of traits across different treatments and environments and opening new opportunities in wheat breeding for biotic and abiotic studies (Rimbert et al., 2018). The International Centre for Agricultural Research in Dry Areas (ICARDA) and the International Maize and Wheat Improvement Centre (CIMMYT) have both played pivotal roles in the development of high-yielding, abiotic stress-tolerant, disease-resistant, higher end-use quality, and widely adaptive global wheat germplasm (Wu et al., 2021).

An improved wheat line 130675 from the International Winter Wheat Breeding Program (IWWIP) (Avd/Vee#1//1-27-6275/Cf 1770/3/MV171-C-17466) selected from the Facultative and Winter Wheat Observation Nursery (FAWWON 2013-2014) possesses several desirable traits, including yield and early maturity, and showed APR to stripe rust in multiple field trials in Turkey. However, it was susceptible to *PstS2* and *Warrior* races at the seedling stage, indicating typical APR for both races. The resistance to stripe rust of the wheat line 130675 has not been characterized. Therefore, the current study aimed to map and characterize adult plant stripe rust resistance loci in the doubled haploid (DH) population derived from a cross between wheat line 130675 and universal stripe rust-susceptible variety Avocet S.

## Material and Methods

### Plant Material and Pathogen

The panel of 190 DH lines from the cross of an improved IWWIP line 130675 (Avd/Vee#1//1-27-6275/Cf 1770/3/MV171-C-17466) and Avocet S (AvS) were evaluated for adult plant stripe rust resistance. The DH lines derived from the F1 generation (F1DH) were developed using the wheat maize hybridization protocol (Sadasivaiah et al., 1999). The parents were selected due their diverse genetic backgrounds and different levels of stripe rust resistance. The stripe rust isolates *PstS2* and *Warrior* (*PstS7*) were used in artificial field inoculations, and both belonged to *PstS2v27* and *PstS7vWarrior* lineages, and the virulence/avirulence formula of the two races are given in Table 1.

**TABLE 1:** Virulence/avirulence formula for the *PstS2* and *Warrior* pathotypes of *Pst*.

Pathotype	Avirulence formula	Virulence formula
*Warrior* (*PstS7*)	*Yr5*, *Yr10*, *Yr15*, *Yr24*, and *Yr27*	*YrA*, *YrAvS*, *Yr1*, *Yr2*, *Yr3*, *Yr4*, *Yr6*, *Yr7*, *Yr8*, *Yr9*, *Yr17*, *Yr25*, *Yr32*, *YrSp*, *YrSu*, *YrND*, *YrSD*, and *YrTres*
*PstS2*	*Yr1*, *Yr3*, *Yr4*, *Yr5*, *Yr10*, *Yr15*, *Yr17*, *Yr24*, *Yr32*, *YrSp*, *YrND*, *YrSD*, and *YrTres*	*YrA*, *YrAvS*, *Yr2*, *Yr6*, *Yr7*, *Yr8*, *Yr9*, *Yr25*, *Yr27*, and *YrSu*

### Field Adult Plant Resistance Assessment

The field experiments were carried out at the precision wheat stripe rust phenotyping platform, Regional Cereal Rust Research Center (RCRRC), Izmir, Turkey, during the cropping seasons 2019, 2020, and 2021. The experiment was laid out as an augmented design with un-replicated test entries and repeated check rows in 12 blocks. Each block contained 16 test entries and seven checks. Thirty seeds from each accession were planted in a 1-m row with 30-cm spacing between the rows. To ensure sufficient inoculum production for disease infection, a mixture of the universally susceptible varieties “Morocco,” “Seri 82,” and “Avocet S” along with the locally susceptible varieties “Bolani,” “Basribey” (also derived from the CIMMYT cross “Kauz”), and “Cumhuriyet 75,” “Kunduru,” “Kasifbey,” and “Gonen” were planted as spreader after every 20 rows, as well as spreader rows bordering the nurseries. The experiments were managed as per the standard local agronomic practices during the crop season.

*PstS2* and *Warrior (PstS7)* pathotypes of stripe rust preserved at RCRRC were multiplied using susceptible variety AvS, and the freshly collected urediniospores were used for field inoculations. The DH panel along with the spreader rows bordering the experiment was artificially sprayed with a mixture of the two races in talcum powder using a backpack sprayer at the seedling, tillering, and booting stages. The field was irrigated through a mist irrigation system.

Field scoring started when disease severity reached 100% on the susceptible checks, “Morocco” and AvS. Adult plant responses were recorded three times at 10-day intervals for the major infection types resistant (R), moderate resistant (MR), moderate (M), moderate susceptible (MS), and susceptible (S) (Roelfs et al., 1992), and the disease severities (0-100%) following the modified Cobb’s scale (Peterson et al., 1948). All the three recordings were averaged, and the coefficients of infection (CI) were calculated. The CIs were calculated by multiplying the constant values of the infection types and disease severity. The constant values of infection types were used as R = 0.2, MR = 0.4, M = 0.6, MS = 0.8, and S = 1 (Saari and Wilcoxson 1974; Stubbs et al., 1986).

### DNA Extraction and Genotyping

Genomic DNA was extracted from fresh leaves collected from three individual 10-day-old seedlings using a modified cetyltrimethylammonium bromide (CTAB) method (Doyle and Doyle 1987). The seedling leaves were collected in labeled Eppendorf tubes and stored in an Ultra freezer at −80°C for subsequent DNA extraction. The leaf samples were grounded using a mortar in liquid nitrogen until a fine powder was obtained, and 0.1 g of the powdered leaf samples were used for DNA extraction using the CTAB method (Doyle and Doyle 1987). The extracted DNA was dissolved in 100 µl Tris–EDTA (TE) buffer. The samples were analyzed on 1% agarose gel for the purity test and quantified with a biophotometer (BioPhotometer, Eppendorf). The DNA samples were then kept at −80°C. The extracted DNA samples of the DH panel and two parental lines were sent to Diversity Arrays Technology Pty Ltd. (Canberra, Australia, http://www.DiversityArrays.com/) for genotyping. The genotypic data obtained for 172 DH lines including parents were filtered, and markers with > 10% missing data and < 0.1% minor allele frequency were eliminated and not used in the subsequent analysis.

### Statistical Analysis

Descriptive statistics and analysis of variance (ANOVA) were performed using the R package “AugmentedRCBD”. Broad-sense heritability was estimated as the ratio of genetic variance (σ^2^_g_) to phenotypic variance (σ^2^_g_ + σ^2^_ε_), where σ^2^_ε_ represents error variance and is represented as follows:

$$H^{2}=\frac{\sigma_{g}^{2}}{\sigma_{g}^{2}+\sigma_{\varepsilon }^{2}}.$$

### Linkage Map Construction and QTL Mapping

The marker genetic data were used to construct the linkage map using the software QTL Ici-Mapping software V4.2. The Kosambi function was used to calculate the genetic distances between the markers (Kosambi 1944). The stripe rust resistance QTLs were estimated in the DH population based on the CI of the 3 years. The composite interval mapping (CIM) method was used for the detection of QTL using QTL Ici-Mapping software V4.2. The threshold value for the logarithm of odds (LOD) score was calculated after running a permutation test of 1,000 runs and was 2.1, 2.0, and 2.4 for 2019, 2020, and 2021 experiments, respectively, with a walking step of 1 cM (Van Ooijen 1999). The QTLs were also reported significant at a threshold of 2.0 if found in multiple years. The effects of QTLs were calculated as the proportion of phenotypic variance explained by the QTL. The genomic locations of the significant QTL were indicated using the software Map Chart V2.3.

### Gene Annotation

The candidate genes with their putative proteins/enzymes were predicted within the interval of 500 kb upstream and downstream from the closest significant markers using Ensembl, a plant database available at http://plants.ensembl.org/Triticum_aestivum/Info/Index, and the International Wheat Genome Sequencing Consortium (IWGSC) *RefSeq v1.1* annotations (Appels et al., 2018) available at https://wheat-urgi.versailles.inra.fr/Seq-Repository/Annotations. The nearby genes in the linkage regions of significant markers with putative functions that could be related to the trait were selected as candidates.

## Results

### Field Assessment of Resistance

**FIGURE 1:**
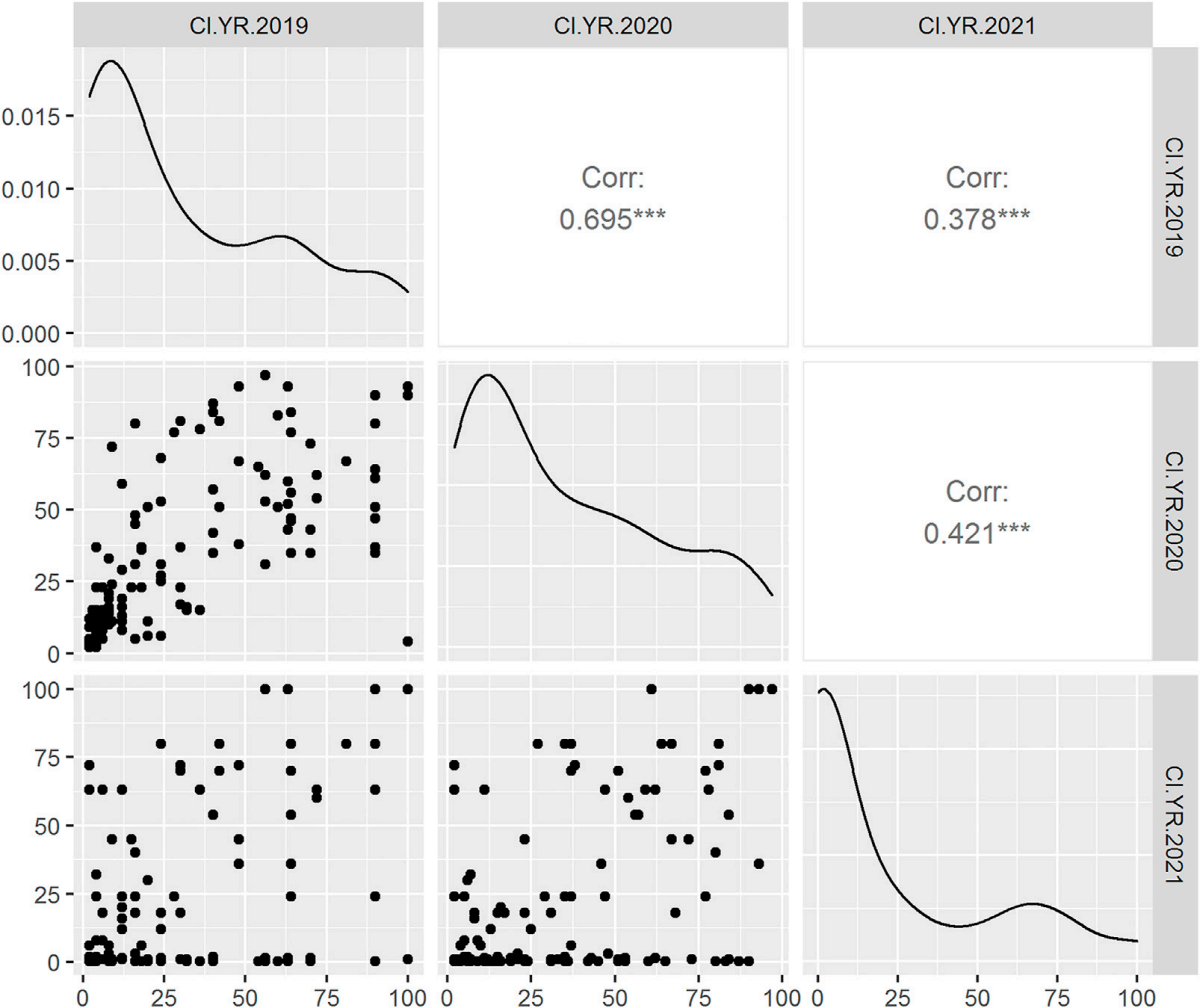
Scatter plot (lower triangle) with the distribution of phenotypic data during 2019, 2020, and 2021 field years from left to right anticlockwise, respectively; density plot (diagonal line) and Pearson correlation analysis (upper triangle) between the 3 years of DH population in field condition. The *X*-axis and *Y*-axis represent the stripe rust coefficient of infection (CI).

In adult plant assessment, the estimates of genetic variance identified significant differences among the DH lines (Table 2). A small variation was observed in the disease severity scores of the tested accessions during the 3 years as in 2021, and the data were more skewed toward the resistance side (Figure 1). Overall, during the 3 years 34.9, 36.9, and 47.92% of the DH lines showed resistance response. The mean values of CI for 2019 and 2020 were 34.5 and 37, respectively, whereas in 2021, the mean value dropped to 21.6. The mean values for the parents ranged from 0.2 to 20 for the resistant parent, while 79 to 90 for the susceptible parent. The broad-sense heritability was 87.04, 91.14, and 77.18 for 2019, 2020, and 2021, respectively. Significant positive correlations were found between the 3 years of field data. The highest correlation (0.69) was found between the CIs from 2019 and 2020, whereas the lowest CI correlation (0.38) was observed between 2019 and 2021 (Figure 1).

**TABLE 2:** Basic statistics of adult plant response of bread wheat DH lines against *PstS2* and *Warrior* pathotypes of stripe rust, estimates of variance components, and broad-sense heritability.

Parameter	DH-2019	DH-2020	DH-2021
Minimum	2	1	0.2
Mean	34.5	37	21.6
Maximum	100	97	100
σ^2^_G_	875.49^***^	742.35^***^	523.41^***^
σ^2^_E_	130.25	72.1	391.88
σ^2^_P_	1,005.74	814.45	915.29
Heritability	87.04	91.14	77.18

^***^Significance at 1% probability level.

σ^2^_G_ = estimates of genotypic variance.

σ^2^_E_ = estimates of error variance.

σ^2^_P_ = estimates of phenotypic variance.

### Linkage Map and Identification of QTLs for Adult Plant Resistance to Stripe Rust in the DH Population

**FIGURE 2:**
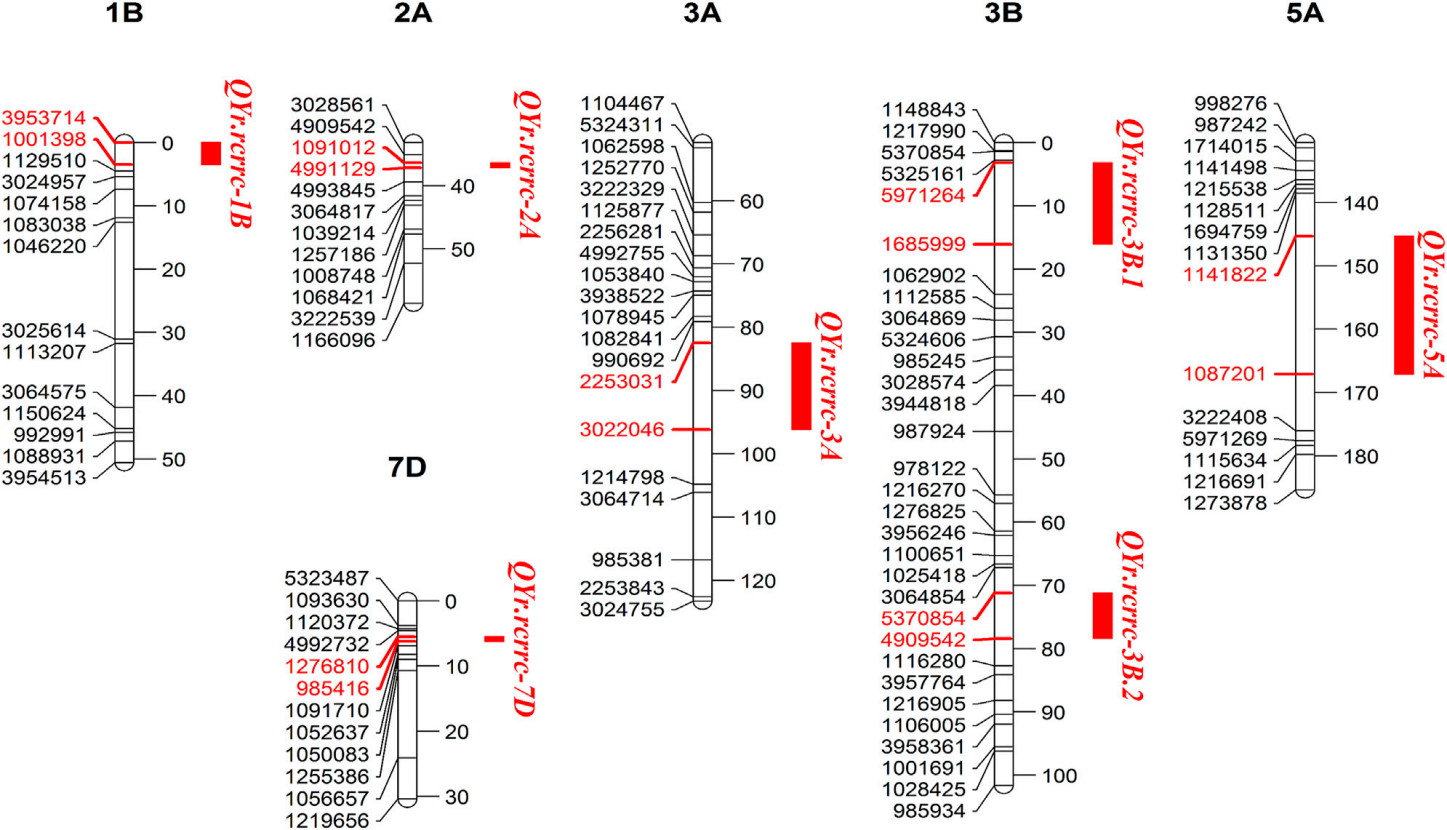
Segments of genetic linkage maps of QTL conferring adult plant stripe rust resistance. Single-nucleotide polymorphism (SNP) markers are shown on the left and their genetic positions (cM) are on the right of chromosomes. The region containing the QTL is indicated by a vertical bar on the right and followed by the name of the QTL. The markers in red are associated with the QTL.

After filtering for quality parameters such as missing data and segregation distortion, a set of 590 skeleton SNP markers were used to construct a linkage map for the 130675 × AvS DH population. The markers covered the whole genome and were divided into 28 linkage groups, marker order in the linkage group was generally in agreement with the published consensus map (Li et al., 2015). Genomes A, B, and D had 244 (41.33%), 237 (40.17%), and 109 (18.47%) markers, respectively, and the total map length was 2,232 cm. Composite interval mapping identified 10 QTLs in 3 years on seven genomic regions across the genome for resistance to yellow rust (Yr) at the adult plant growth stage; the QTLs’ were named *QYr.rcrrc.1B*, *QYr.rcrrc.2A*, *QYr.rcrrc.3A*, *QYr.rcrrc.3B.1*, *QYr.rcrrc.3B.2*, *QYr.rcrrc.5A*, and *QYr.rcrrc.7D*. Out of these 10 QTLs, three were detected in the 2019 and 2021 field years, while four were detected in 2020. The QTLs were detected on seven genomic regions in chromosomes 1B, 2A, 3A, 3B, 5A, and 7D (Figure 2).

The phenotypic variance explained by an individual QTL ranged from 3.4 to 20.6%. Two stable QTLs on chromosome 3B, that is, *QYr.rcrrc.3B.1* and *QYr.rcrrc.3B.2* were detected in multiple years and contributed 5.2–19.8% toward phenotypic variation. The QTL that explained a phenotypic variance of more than 10% was considered as a major QTL. All QTLs were contributed by the resistant parent 130675 except one on chromosome 3A, which was contributed by the susceptible parent AvS (Table 3).

**TABLE 3:** Quantitative trait loci (QTLs) associated with adult plant stripe rust resistance in DH population in different environments.

Year	QTL	Flanking marker	Chromosome	LOD	PVE (%)	Resistance source	Previous QTL/gene	Reference
**2019**	*QYr.rcrrc-1B*	*3953714–1001398*	1B	2.49	3.42	130675	*Yr29/Lr46*; *QYr.sun-1B_Wollaroi*; *QYr.ucw-1B (IWA3892)*	William et al. (2003); Bansal et al. (2014); Maccaferri et al. (2015)
	*QYr.rcrrc-3B.1*	*5971264–1685999*	3B	3.7264	5.20	130675	*Yr4*; *Yr57*; *QYr-3B_Opata85*; *QYr.tam-3B_Quaiu*	Singh et al. (2000); Suenaga et al. (2003); McIntosh et al. (2014); Basnet et al. (2014)
	*QYr.rcrrc-3B.2*	*5370854–4909542*	3B	2.2211	10.63	130675	*QYr.cim-3B_Pastor* ; *QRYr3B.2*; *SNP1863248*	Rosewarne et al. (2012); Jighly et al. (2015); Tehseen et al. (2021)
**2020**	*QYr.rcrrc-3A*	*2253031–3022046*	3A	2.3805	3.68	Avocet S	*QYr.cim-3A_Avocet*	Rosewarne et al. (2008)
	*QYr.rcrrc-3B.1*	*5971264–1685999*	3B	8.2825	12.84	130675	*Yr4*; *Yr57*; *QYr-3B_Opata85*; *QYr.tam-3B_Quaiu*	Singh et al. (2000); Suenaga et al. (2003); McIntosh et al. (2014); Basnet et al. (2014)
	*QYr.rcrrc-3B.2*	*5370854–4909542*	3B	2.6826	11.58	130675	*QYr.cim-3B_Pastor* ; *QRYr3B.2*; *SNP1863248*	Rosewarne et al. (2012); Jighly et al. (2015); Tehseen et al. (2021)
	*QYr.rcrrc-7D*	*1276810–985416*	7D	3.5807	5.04	130675	Novel	Current study
**2021**	*QYr.rcrrc-2A*	*1091012–4991129*	2A	11.6495	20.63	130675	Novel	Current study
	*QYr.rcrrc-3B.2*	*5370854–4909542*	3B	5.765	19.83	130675	*QYr.cim-3B_Pastor* ; *QRYr3B.2*; *SNP1863248*	Rosewarne et al. (2012); Jighly et al. (2015); Tehseen et al. (2021)
	*QYr.rcrrc-5A*	*1141822–1087201*	5A	2.5222	3.81	130675	*Yr34*; *QYrdr.wgp-5AL (IWA2646)*; *QYr-5A_Opata85*; *QYr.cim-5AL*_Pastor	Chen et al. (2021); Hou et al. (2015); Boukhatem et al. (2002); Rosewarne et al. (2012)

### QTL Region Physical Positions and Candidate Gene Prediction

The alignment of significant QTL markers with reference genome confirmed their physical positions according to chromosome assignments (Table 4). The largest physical distance of 53.7 Mb spanned between the flanking markers of *QYr.rcrrc-5A*. The *QYr.rcrrc-5A* also spanned a large interval on the genetic map compared with other QTLs (Figure 2). The physical distances between *QYr.rcrrc-2A*, *QYr.rcrrc-3B.1*, and *QYr.rcrrc-7D* were 1.1, 4.7, and 0.6 Mb, respectively. The expressed genes between the flanking markers of the QTL were identified using the BLASTn searches from the flanking markers sequences (Table 5). The high confidence genes which were previously reported to be associated with disease resistance were selected as candidate genes.

**TABLE 4:** Physical position of the SNP markers that flank the quantitative trait loci (QTLs).

QTL	Flanking markers	Physical position (Mb)^a^
*QYr.rcrrc-1B*	*3953714*	667.138
	*1001398*	--^b^
*QYr.rcrrc-2A*	*1091012*	755.80
	*4991129*	756.91
*QYr.rcrrc-3A*	*2253031*	655.66
	*3022046*	701.93
*QYr.rcrrc-3B.1*	*5971264*	5.58
	*1685999*	10.35
*QYr.rcrrc-3B.2*	*5370854*	580.06
	*4909542*	--
*QYr.rcrrc-5A*	*1141822*	612.914
	*1087201*	666.70
*QYr.rcrrc-7D*	*1276810*	104.88
	*985416*	104.21

a Physical position was mapped by aligning the sequence against Chinese Spring assembly from the International Wheat Genome Sequencing Consortium (IWGSC) RefSeq ver. 1.0.

b No hit.

**TABLE 5:** List of candidate genes for each QTL with putative proteins/enzymes.

QTL	Gene	Chromosome	Protein/enzyme
*QYr.rcrrc.1B*	*TraesCS1B01G447000*	1B	NB-ARC domain
*QYr.rcrrc.2A*	*TraesCS2A01G547500*	2A	Protein kinase domain
*QYr.rcrrc.3A*	*TraesCS3A01G411400*	3A	Leucine-rich repeat domain
*QYr.rcrrc.3B.1*	*TraesCS3B01G012400*	3B	F-box domain
*QYr.rcrrc.3B.2*	*TraesCS3B01G368000*	3B	Leucine-rich repeat domain
*QYr.rcrrc.5A*	*TraesCS5A01G500400*	5A	NAC domain protein
*QYr.rcrrc.7D*	*TraesCS7D01G192900LC*	7D	Leucine-rich repeat domain

## Discussion

Stripe rust is a devastating disease of wheat and could result in 100% yield loss under high disease pressure (Manickavelu et al., 2016). Historically, it was considered a disease in wet and cool climates but with the emergence of new races adapted to high temperatures the disease has sporadically spread to areas that were once considered unsuitable for its growth and disease development (Hovmøller et al., 2016; Ali et al., 2017). The most effective strategy to manage the continuous appearance of new stripe rust races is genetic resistance and the development of lines harboring both minor and major genes (Chen et al., 2014). The plant breeders tend to stack multiple different traits in elite backgrounds; therefore, breeding for one trait is not always simple and even in the presence of highly resistant germplasm breeders do not necessarily always utilize it due to the undesirable linkage drag associated with resistance locus. Hence, to circumvent the potential linkage drag, the breeders focus on identifying and mapping resistance genes from elite breeding lines with accumulated favorable morphological and agronomic traits. The current study used an advanced breeding line with a distinct resistance response to stripe rust from the IWWIP breeding program.

Overall, 10 QTLs in seven genomic regions across the three environments (years) were detected in the current study. The phenotypic variance explained by the QTL ranged from 3.4 to 20.6% confirming their significant effects in reducing stripe rust severity. The significant QTLs detected in the study were compared with the previously published stripe rust known genes and QTL based on their chromosome location, physical position, pedigree, linked markers, and rust resistance.

The *QYr.rcrrc-1B* detected in the current study on chromosome 1B overlapped the several previously reported *Yr* QTL and an APR gene *Yr29* (William et al., 2003; Bansal et al., 2014; Maccaferri et al., 2015). The pleiotropic locus *Yr29/Lr46/Sr58* on chromosome 1B has been widely used in breeding programs around the world, including CIMMYT wheat germplasm (Gebrewahid et al., 2020). The *Yr29/Lr46/Sr58* locus is associated with a wide-spectrum resistance level explaining 2.9–74.5% of the phenotypic variation in different bi-parental mapping populations and under different environmental trials (Zhang et al., 2019). The stripe rust *Yr29* is a slow rusting adult plant resistance gene and its effect decreases with the increase in the inoculum load. This could be the plausible reason why *QYr.rcrrc-1B* was detected only in the 2019 crop year. *QYr.rcrrc-1B* is most likely the *Yr29/Lr46/Sr58* complex.

The *QYr.rcrrc-2A* detected in the current study does not correspond to any of the previously identified *Yr* QTL and/or genes. A seedling resistant gene *Yr1* is also located on the long arm of chromosome 2A; however, according to the physical position of *Yr1*, the gene and the *QYr.rcrrc-2A* are 15.4 Mb apart at the distal end. *QYr.wpg-2A.6 (IWA966)* a minor effect APR QTL was also reported on chromosome 2A and is the closest QTL to *QYr.rcrrc-2A* and the two are 6.79 Mb apart (Naruoka et al., 2015). Since *QYr.rcrrc-2A* is a major QTL and does not overlap with any of the previously reported *Yr* QTL/gene; furthermore, the closest gene to *QYr.rcrrc-2A* is *Yr1* which is a seedling resistant gene and is ineffective against the *Warrior* (*PstS7*) race used in the study. Therefore, based on the physical locations of the close-by QTL and resistance pattern of the nearby genes, the QTL *QYr.rcrrc-2A* is considered novel.

*QYr.rcrrc-3A* was found significant to the stripe rust resistance in the field. The locus overlapped a previously reported *Yr* QTL *QYr.cim-3A_Avocet* (Rosewarne et al., 2008). The *QYr.cim-3A_Avocet* was found in a RIL population derived from a cross between AvS and Pastor, the two QTLs expressed similar total phenotypic variation and the source of resistance in both the populations was cultivar AvS. Therefore, based on the physical overlapping positions, a similar effect of QTL, and sources of resistance in both studies, it was concluded that both *QYr.rcrrc-3A* and *QYr.cim-3A_Avocet* represent the same genomic region.

Two stable QTLs such as *QYr.rcrrc-3B.1* and *QYr.rcrrc-3B.2* were detected on the short and long arms of chromosome 3B, respectively. *QYr.rcrrc-3B.1* lies in the same genomic region as the several *Yr* QTLs and genes reported earlier on the short arm of chromosome 3B (Singh et al., 2000; Suenaga et al., 2003; Basnet et al., 2014; McIntosh et al., 2014). The seedling resistance gene *Yr4* is avirulent on one of the pathotypes that is *PstS7* used in the study for artificial inoculation. Based on the physical position and virulence/avirulence pattern it is likely that *QYr.rcrrc-3B.1* represent is linked to *Yr4*; however, further studies are required to confirm the relationship as some studies have reported a different APR locus in the absence of *Yr4* (Buerstmayr et al., 2014). *QYr.rcrrc-3B.*2, the second stable QTL detected in all three field experiments was found on the long arm of chromosome 3B and overlapped the previously reported *QYr.cim-3B_Pastor*, *QRYr3B.2*, and *SNP1863248* (Rosewarne et al., 2012; Jighly et al., 2015; Tehseen et al., 2021). The three previously reported QTLs conferred APR; therefore, it is likely that *QYr.rcrrc-3B.*2 is linked to these QTLs.

A minor effect of QTL *QYr.rcrrc-5A* was detected on the long arm of chromosome 5A and overlapped the same genomic region previously reported to be linked with several APR and high-temperature adult plant (HTAP) *Yr* QTL (Boukhatem et al., 2002; Rosewarne et al., 2012; Hou et al., 2015). An APR gene with a moderate level of resistance is also located in the same genomic location (Chen et al., 2021). Since *QYr.rcrrc-5A* is a minor effect on QTL and *Yr34* also shows moderate resistance it is likely that *QYr.rcrrc-5A* is linked with *Yr34*; however, further genetic analysis is required to confirm the relationship as no source of resistance with *Yr34* was used in the differential set for race typing of the pathotypes used in the current study.

Two *Yr* resistant genes and a seedling resistance marker are previously reported on chromosome 7D (Maccaferri et al., 2015; Bulli et al., 2016; Tehseen et al., 2021). However, the locus *QYr.rcrrc-7D* detected in the current study is outside the genomic regions of the two genes and the QTL. The approximate distance between *QYr.rcrrc-7D* and the gene *Yr33* and the seedling resistant locus *QYr.7D_seedling* is 33Mb and 17 Mb, respectively. Thus, based on the physical distances *QYr.rcrrc-7D* is a novel QTL region.

Regarding the predicted proteins in the current study, the candidate genes include NB-ARC domain proteins, which are involved in pathogen recognition and subsequent activation of plants’ defense mechanisms (Van Ooijen 1999; Van Ooijen et al., 2008; Steele et al., 2019); protein kinase domain proteins, which modify other proteins and are vital in several signaling and regulatory pathways in addition to apoptosis and cell division (Brueggeman et al., 2008); and leucine-rich repeats (LRR), which play a vital role in plants’ defense mechanism and are typically annotated to resistance genes (Jones and Jones 1997; Yuan et al., 2018), F-box domain proteins; they are involved in plant vegetative and reproductive growth and development. These proteins are reported to regulate cell death and defense when the pathogen is recognized in the tobacco and tomato plant (van den Burg et al., 2008), and NAC domain proteins which are involved in several processes, including the formation of secondary walls, senescence, and abiotic and biotic stresses (Puranik et al., 2012; Ng et al., 2018; Yuan et al., 2019). All candidate genes have been previously reported to play role in the plant’s defense mechanism; therefore, it is highly likely that they could be one of the candidate genes for stripe rust resistance. However, these putative candidate proteins should be used with caution as they are not the only proteins found within the confidence intervals of the linked markers but are the ones that have been reported to be involved in plant defense and disease and/or stress resistance mechanisms.

Marker-assisted breeding (MAB) is a valuable tool and is being utilized in many breeding programs around the world for different kinds of crops. MAB allows successful introgression of biotic and abiotic stress-resistant genes in high-yielding susceptible backgrounds (Ren et al., 2012). Therefore, detection of significant and tightly linked markers is desirable, which can be converted into breeder-friendly markers to be utilized in the breeding programs through MAB. In this study, we identified seven QTLs associated with APR to stripe rust across environments including *QYr.rcrrc-1B*, *QYr.rcrrc-2A*, *QYr.rcrrc-3A*, *QYr.rcrrc-3B.1*, *QYr.rcrrc-3B.2*, *QYr.rcrrc-5A*, and *QYr.rcrrc-7D*, and they were closely linked to SNP markers *3953714*, *1091012*, *2253031*, *5971264*, *5370854*, *1141822*, and *1276810*, respectively. With new extensive research and cloning of APR genes, the overall function of the APR genes is better understood. However, the durability of any APR gene or the combination of APR genes is still a mystery and is based on prediction and time (Lowe et al., 2011). Nevertheless, the QTL reported in the current study particularly *QYr.rcrrc-2A* and *QYr.rcrrc-7D* were new QTL for APR to stripe rust. They should enhance the genetic basis of resistance to stripe rust, and their closely linked markers can be converted into breeder-friendly markers and utilized in MAB and stacking of multiple APR genes in common wheat backgrounds for durable resistance to stripe rust.

## Data Availability

The datasets presented in this study can be found in online repositories. The names of the repository/repositories and accession number(s) can be found in the article/Supplementary Material.
